# Subinhibitory Concentrations of Amoxicillin, Lincomycin, and Oxytetracycline Commonly Used to Treat Swine Increase *Streptococcus suis* Biofilm Formation

**DOI:** 10.3389/fmicb.2018.02707

**Published:** 2018-11-06

**Authors:** Ursula Waack, Tracy L. Nicholson

**Affiliations:** ^1^United States Department of Energy, Oak Ridge Institute for Science and Education, Oak Ridge, TN, United States; ^2^United States Department of Agriculture, National Animal Disease Center, Agricultural Research Service, Ames, IA, United States

**Keywords:** *Streptococcus suis*, biofilm, subinhibitory concentrations, antibiotics, swine, agriculture

## Abstract

*Streptococcus suis* is a bacterial swine pathogen with a significant economic burden. It typically colonizes the tonsil and nasal cavity of swine causing a variety of symptoms ranging from asymptomatic carriage to lethal systemic disease. A key barrier toward the development of improved vaccines or interventions for *S. suis* infections is a gap in our understanding of the mechanisms contributing to persistence in the host, in which colonized pigs continue to shed and transmit *S. suis.* We hypothesized that exposure to sub-MICs of antibiotics commonly used by the swine industry would increase the biofilm capacity of *S. suis* strains. Using a 96-well plate MIC protocol, we experimentally determined the MIC for each of 12 antibiotics for a virulent strain of *S. suis* strain that consistently formed biofilms using a standard crystal violet assay. Using this static biofilm assay, we demonstrated that sub-MICs of bacitracin, carbadox, chlortetracycline, enrofloxacin, gentamicin, neomycin, sulfadimethoxine, tiamulin, and tylosin did not increase *S. suis* biofilms. In contrast, we demonstrated that sub-MICs of amoxicillin, lincomycin, and oxytetracycline increased overall biofilm formation under both static and flow conditions. The biofilm formation of 11 additional clinical isolates were measured using the relevant concentrations of amoxicillin, lincomycin, and oxytetracycline. Eight of the eleven strains increased the biofilm formation with lincomycin, seven with amoxicillin, and three with oxytetracycline. Collectively, our data demonstrate that exposure to sub-MICs of these commonly used antibiotics contributes to increased biofilm formation of *S. suis*, thereby potentially increasing survival and persistence within the respiratory tract of swine.

## Introduction

Respiratory disease in swine places a significant economic burden on producers (USDA, 2015a). Common causes of respiratory disease include influenza, porcine respiratory and reproductive syndrome virus, and *Streptococcus suis* (USDA, 2015a). *S. suis* is a Gram positive pathogen that colonizes the respiratory tract of pigs. Infected animals may remain asymptomatic carriers or develop clinical signs of disease including pneumonia, meningitis, and arthritis. Asymptomatic carriers are considered a transmission reservoir and can serve as a source of infection for other animals. A survival method commonly used by bacteria is the formation of biofilms that are increasingly recognized as important contributors to chronic or persistent infections (Costerton et al., 1999; Donlan and Costerton, 2002).

Biofilms are bacterial communities embedded in an extracellular matrix composed of carbohydrates, extracellular DNA (eDNA), and/or proteins and may be single-species or multi-species in nature (Hall-Stoodley et al., 2004; de la Fuente-Nunez et al., 2013). Growth in biofilms may increase the ability of bacteria to survive external challenges such as antimicrobials, host immune factors, environmental stressors such as shear forces, and changes in nutrient availability (Hall-Stoodley et al., 2004; Hall and Mah, 2017). For example, a study by Ma et al. (2017) showed that *S. suis* biofilms can decrease neutrophil extracellular traps (NET) formation and assist in evading the host’s immune system. Additionally, bacteria in biofilms have demonstrated an increase in both persistence and infectivity. Chronic respiratory infections such as recurrent middle ear infections and bacterial infections in cystic fibrosis patients have been caused by biofilm-forming strains of bacteria such as *Pseudomonas aeruginosa* (Hall-Stoodley et al., 2004; Nazzari et al., 2015). Bacteria in biofilms are able to withstand therapeutic interventions and serve as a reservoir for future infections. *Vibrio cholerae* cells from a biofilm required a lower infectious dose to colonize than planktonic cells suggesting a hyperinfectious phenotype (Tamayo et al., 2010). This occurrence is not limited to human-associated infections as biofilms routinely contribute to common veterinary-associated diseases such as pneumonia and mastitis (Olson et al., 2002; Fox et al., 2005).

Not only does growth inside a biofilm increase the antibiotic resistance of the bacteria within the biofilm, but sub-minimal inhibitory concentrations (sub-MICs) have been shown to increase biofilm formation in certain bacterial species (Kaplan, 2011; Kaplan et al., 2012; Mlynek et al., 2016). Only a few studies exist evaluating *S. suis* biofilm formation and only three studies have been published regarding the effect of sub-MICs of antibiotics on *S. suis* biofilm formation (Zhao et al., 2015; Wang et al., 2016; Yang et al., 2016). In addition, all three of the studies have focused primarily on common antibiotics used to treat *S. suis* disease in humans. Erythromycin (Zhao et al., 2015), tylosin (Wang et al., 2016), and azithromycin (Yang et al., 2016) have all been shown to inhibit biofilm formation of *S. suis* strain ATCC 700794 at sub-MIC levels. All three of these antibiotics are macrolides and only one, tylosin, is used to treat swine. While *S. suis* is a zoonotic pathogen capable of causing severe human infections, pigs are the natural hosts and reservoir of *S. suis*. A complete understanding of the factors contributing to persistent colonization combined with effective treatment strategies is critically needed to decrease the burden of *S. suis* disease for both human and animal health. We hypothesized that sub-MICs of important antibiotics used in the swine industry would increase the formation of *S. suis* biofilm. To address this hypothesis, we measured the development of *S. suis* biofilms upon exposure to sub-MICs of antibiotics under both static and flow-cell conditions.

## Materials and Methods

### Bacterial Strains and Growth Conditions

*S. suis* strain ISU1606 was isolated from a pig displaying neurological symptoms consistent with *S. suis* disease and obtained from the Iowa State University College of Veterinary Medicine, Veterinary Diagnostic Laboratory, Ames, IA. Virulence was subsequently confirmed following intranasal challenge of caesarean-derived, colostrum-deprived (CDCD) pigs (unpublished data). BHI + 5% horse serum + 100 μg/mL nicotinamide adenine dinucleotide (NAD) was used for growth throughout the study and the strain was incubated at 37°C with 5% CO_2_. Additional clinical *S. suis* isolates acquired between 2015 and 2017 were obtained from the University of Minnesota Veterinary Diagnostic Laboratory, Saint Paul, MN.

### Antimicrobial Susceptibility Testing

A standard broth dilution method was used to determine the MIC of each antibiotic against ISU1606. Antibiotics were diluted twofold in a 96-well flat bottom plate (Corning, Sigma-Aldrich, Darmstadt, Germany) in media. Overnight cultures of ISU1606 were diluted to an OD_600_ of 0.1 and added to each well. The total volume in each well was 100 μL with two wells for every antibiotic concentration. Plates were then incubated under stationary conditions at 37°C and absorbance at OD_600_ was measured after 18 h. incubation. Media only and ISU1606 only wells were used as negative and positive controls, respectively. At least three independent experiments with three technical replicates in each experiment for antibiotic were performed. MIC was determined as the lowest concentration of antibiotic that prevented growth. If ISU1606 was determined to be resistant to an antibiotic, a therapeutic concentration was used for all subsequent experiments. Amoxicillin, Bacitracin, Carbadox, Chlortetracycline, Lincomycin, Neomycin, Oxytetracyline, and Tylosin were obtained from Sigma-Aldrich (St. Louis, MO, United States). Enrofloxacin was obtained from Norbrook Laboratires (Newry, Northern Ireland). Gentamicin was obtained from Invitrogen (Carlsbad, CA, United States). Sulfadimethoxine was obtained from Zoetis (Kalamazoo, MI, United States). Tiamulin was obtained from Novartis Animal Health US (Greensboro, NC, United States).

### Growth Kinetics

Kinetic growth of cultures were measured using a Bioscreen C Automated Microbiology Growth Curve Analysis System (Growth Curves USA, Piscataway, NJ, United States). Briefly, overnight cultures of ISU1606 were diluted to an OD_600_ of 0.1. The appropriate concentration of antibiotic was added to the culture. Hundred microliter of culture was added to each well and four wells were used for every condition tested. Plates were then incubated with gentle agitation at 37°C and the OD_600_ of each well was measured every 30 min. At least four independent experiments with three technical replicates in each experiment for antibiotic were performed.

### Microtiter Plate Assay for Static Biofilm Formation

A static biofilm assay was performed using the standard crystal violet method as reported earlier (Nicholson et al., 2013). Briefly, overnight cultures of ISU1606 were diluted to an OD_600_ of 0.1. Antibiotics were added to the cultures at concentrations experimentally determined from antimicrobial susceptibility tests. Cultures were incubated in a flat-bottomed 96-well plate (Corning, Sigma-Aldrich, Darmstadt, Germany) for 24 h at 37°C. After incubation, the OD_600_ was measured in all cultures to determine growth. The supernatant and any unadhered bacteria were aspirated from all cultures and then the wells were washed three times with 200 μL PBS. The wells were stained with 150 μL 0.1% crystal violet for 10 min. After incubation, the dye was removed and wells were washed three times with 200 μL PBS. After the plate had dried, 150 μL of 100% ethanol was added to the wells and allowed to incubate for 15 min. To determine biofilm levels, 125 μL was then transferred to a new plate and the absorbance was measured at OD_538_. At least three independent experiments with three technical replicates in each experiment for antibiotic were performed. Means were compared using an ordinary one-way analysis of variance (ANOVA) with a Dunnett’s multiple comparison post-test. A 5% level of significance (*p* < 0.05) was considered significant.

### Confocal Laser Scanning Microscopy (CSLM) and Image Analysis

Flow-cell biofilm assay, image acquisition, and analysis were performed as previously reported with slight modifications (Nicholson et al., 2017). Overnight cultures of ISU1606 were diluted to an O.D. of 0.1 in fresh media with or without antibiotics. These cultures were used to inoculate ibidi μ-Slide I 0.4 167 Luer sterile single-use tissue culture treated (ibiTreat) flow cells with a channel height of 400 μm (ibidi USA, Madison, WI, United States). The flow cells were incubated for 90 min at room temperature to allow initial bacterial adherence. Following this incubation, flow of media was initiated at a rate of 0.2 mL/min. Biofilms were allowed to grow for 48 h at 37°C. After growth, mature biofilms were stained with 1 μL/mL SYTO9 and 1 μL/mL propidium iodide using the FilmTracer LIVE/DEAD Biofilm Viability Kit (Invitrogen, Thermo Fisher Scientific, Carlsbad, CA, United States). Imaging was performed using a Nikon A1R+ confocal laser scanning microscope with a 20X objective. Images were acquired at 1024 × 1024 pixels using a Z-step of 0.975μm. SYTO9 staining was detected using an excitation wavelength of 487.4 nm and an emission wavelength of 525 nm. Propidium iodide staining was detected using an excitation wavelength of 562 nm and an emission wavelength of 595 nm. Three independent experiments were performed and at least three different images were acquired per experiment. The image acquisition software used was Nikon NIS-Elements AR 4.40 and post-image analysis was performed using Imaris Software (Bitplane, Concord, MA, United States). Means of quantitative parameters were compared using an ordinary one-way analysis of variance (ANOVA) with a Dunnett’s multiple comparison post-test. A 5% level of significance (*p* < 0.05) was considered significant.

## Results

With the aim of concentrating on relevant antibiotics in the swine industry, we choose antibiotics that are predominantly or commonly used to treat any swine disease. In addition, we focused on antibiotics that are typically administered through food or water for two main reasons. First, a common and efficient treatment practice routinely involves administering antibiotics in the food or water when a pig in the shared space exhibits symptoms consistent with a bacterial infection (USDA, 2015b). Subsequently, clinically healthy pigs may come into contact with an antibiotic that is not effective against *S. suis*. Secondly, due to their rooting behavior, pigs have the potential for small amounts of food and/or water to contact areas within their upper respiratory tract, such as nasal passages, where bacteria present there would be exposed to antibiotics. Therefore, we chose to test 12 different antibiotics that were used in at least 10% of all sites surveyed in the USDA 2012 Swine Health and Health Management survey (USDA, 2015b), with 11 antibiotics commonly administered through food and water and one administered through injection (Table 1).

**Table 1:** Experimentally Derived Antimicrobial Susceptibilities for *S. suis* ISU1606.

Antimicrobial agent	MIC (μg/mL)	Antibiotic class
Amoxicillin*^w^*	0.016	Beta-Lactam
Bacitracin*^F^*	1	Polypeptide
Carbadox*^F^*	R	Quinoxaline
Chlortetracycline*^F,W^*	32	Tetracycline
Gentamicin*^W^*	50	Aminoglycoside
Enrofloxacin*^I^*	0.0625	Fluoroquinolone
Lincomycin*^I,F^*	R	Lincosamide
Neomycin*^W^*	6.25	Aminoglycoside
Oxytetracycline*^I,F,W^*	32	Tetracyline
Sulfadimethoxine*^W^*	R	Sulfonamide
Tiamulin*^F,W^*	0.004	Pleuromutilin
Tylosin*^I,F^*	R	Macrolide

*R, resistant; I, Used as injectable antibiotic; F, Used in Feed; W, Used in Water. All antibiotics were utilized by the method indicated in at least 10% of sites (USDA, 2015b).*

Using a standard broth dilution method, we experimentally determined the MICs of these antibiotics toward a virulent clinical *S. suis* isolate, ISU1606 (Table 1). These concentrations were used in all subsequent experiments. If the *S. suis* strain used exhibited resistance to an antibiotic, a concentration equivalent to a typical therapeutic dosage administered through food or water was then used.

**FIGURE 1:**
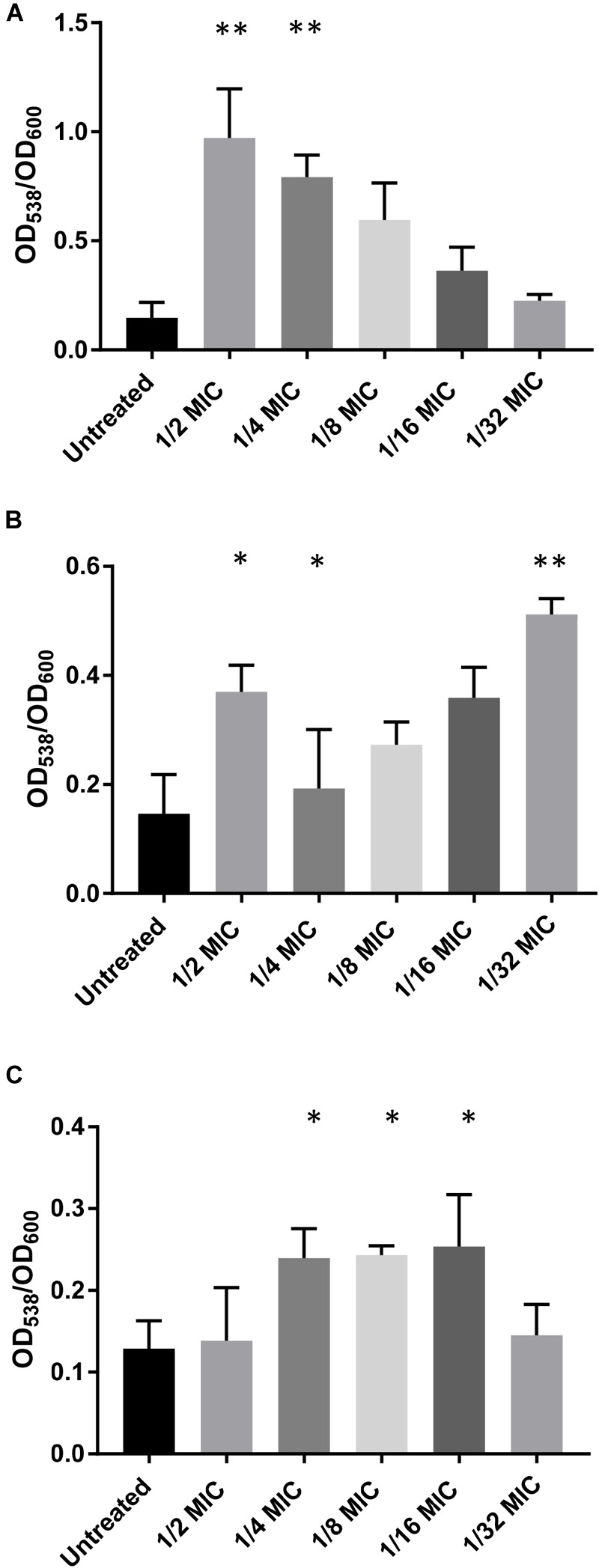
Sub-MICs of specific antibiotics increase biofilms formed by *S. suis*. WT *S. suis* was incubated O/N in a 96-well plate with sub-MICs of commonly used antibiotics. After incubation, the OD_600_ of each well was measured and the plate was stained using a standard crystal violet assay and visualized by OD_538_. All OD_538_ values were normalized by OD_600_ values. Biofilm assay performed with sub-MICs of **(A)** Amoxicillin. **(B)** Lincomycin. **(C)** Oxytetracycline. Values are the means ± standard deviations (error bars) from three independent experiments, with three technical replicates in each experiment. Significant differences were assessed by ordinary one-way ANOVA with Dunnett’s multiple comparison post-test; ^∗^*p* < 0.05, ^∗∗^*p* < 0.01.

**FIGURE 2:**
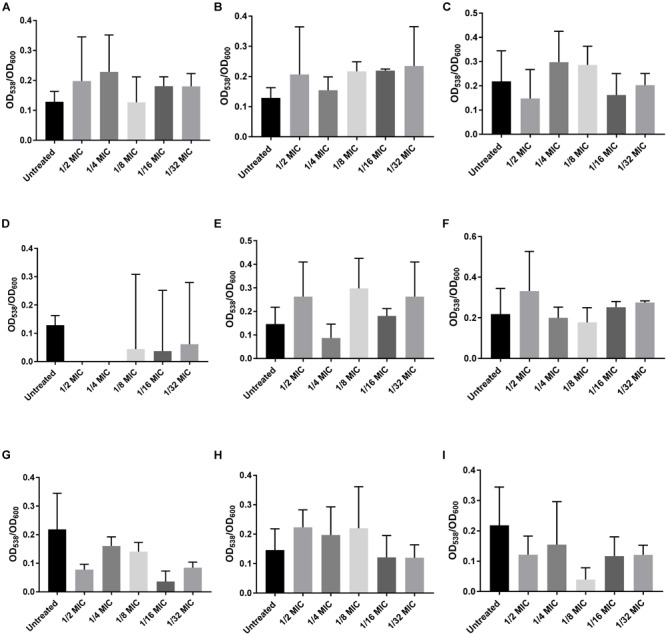
Sub-MICs of specific antibiotics that do not increase biofilms formed by *S. suis*. WT *S. suis* was incubated O/N in a 96-well plate with sub-MICs of commonly used antibiotics. After incubation, the OD_600_ of each well was measured and the plate was stained using a standard crystal violet assay and visualized by OD_538_. All OD_538_ values were normalized by OD_600_ values. Biofilm assay performed with sub-MICs of **(A)** Bacitracin. **(B)** Carbadox. **(C)** Chlortetracycline. **(D)** Enrofloxacin. **(E)** Gentamicin. **(F)** Neomycin. **(G)** Sulfadimethoxine. **(H)** Tiamulin. **(I)** Tylosin. Values are the means ± standard deviations (error bars) from three independent experiments, with three technical replicates in each experiment. Significant differences were assessed by ordinary one-way ANOVA with Dunnett’s multiple comparison post-test.

Of the 12 antibiotics tested, three increased biofilm formation at sub-MICs using a standard crystal violet assay: amoxicillin, lincomycin, and oxytetracycline (Figure 1). Amoxicillin demonstrated a statistically significant increase at ½ and ¼ MICs (Figure 1A). Lincomycin resulted in a significant increase in biofilm formation at ½, ¼, and 1/32 MIC, suggesting a biphasic relationship between antibiotic concentration and biofilm formation (Figure 1B). Oxytetracycline significantly increased biofilm formation at ¼, 1/8, and 1/16 MICs (Figure 1C). The other nine antibiotics (bacitracin, carbadox, chlortetracycline, enrofloxacin, gentamicin, neomycin, sulfadimethoxine, tiamulin, and tylosin) did not increase biofilm formation at any concentration tested (Figure 2).

**FIGURE 3:**
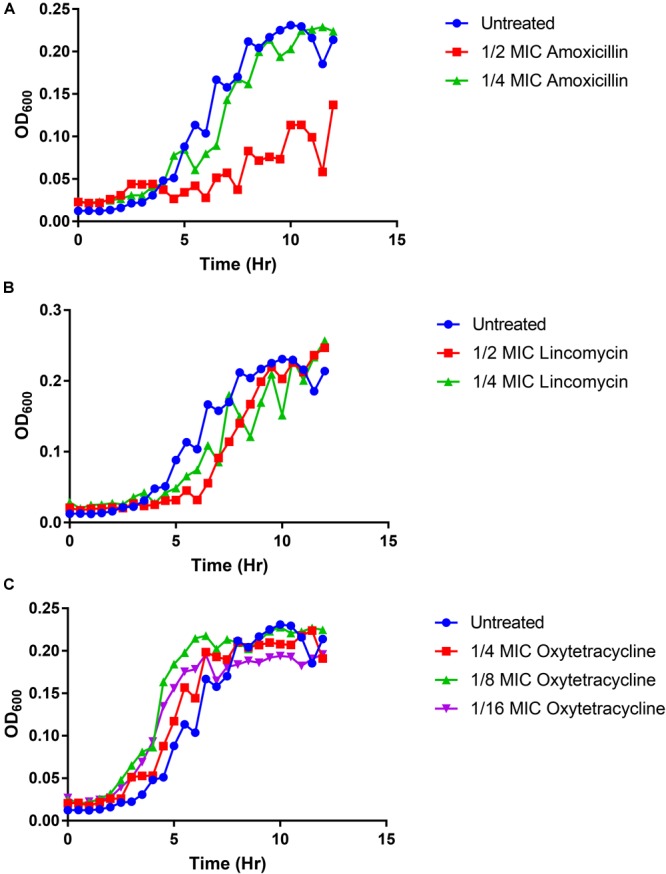
The effect of sub-MICs on *S. suis* kinetic growth. *S. suis* WT strain was grown with or without antibiotics. The OD_600_ was measured every 30 min. *S. suis* with or without sub-MICs of **(A)** Amoxicillin. **(B)** Lincomycin. **(C)** Oxytetracycline. Values are the means from four independent experiments, with three technical replicates in each experiment.

Table 1 lists the MIC of each antibiotic against ISU1606. While all antibiotic concentrations utilized in the biofilm assays had no effect on growth after 18 h, given that sub-MIC concentrations were used, we further tested whether the sub-MIC antibiotic concentration could contribute to kinetic differences in growth and thus present potential confounding factors to our analysis. We measured the kinetic growth of *S. suis* when incubated with the concentration of antibiotics that contributed to an increase biofilm formation (Figure 3). A delay in growth was observed when using ½ MIC of amoxicillin (Figure 3A). Because of this delay, we chose to only use the ¼ MIC of amoxicillin in the subsequent experiments. However, no statistical difference was observed between the untreated wild-type strain and the concentrations of lincomycin or oxytetracycline tested (Figures 3A,B).

**FIGURE 4:**
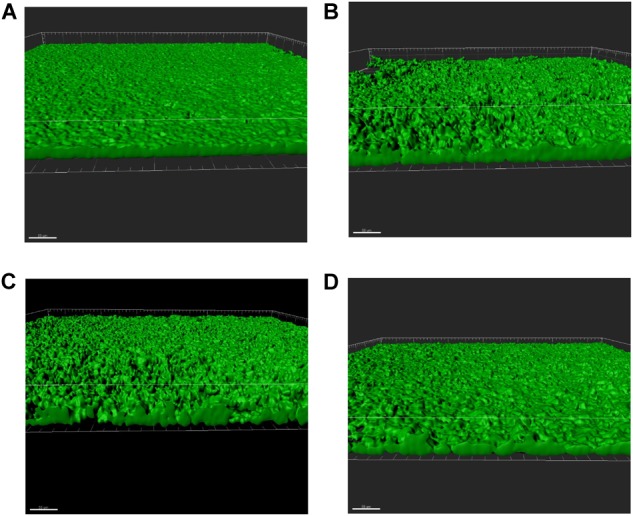
Sub-MICs of specific antibiotics increase biofilm formation. *S. suis* was inoculated into continuous flow chambers in a flow-cell biofilm assay with or without antibiotics. The bacteria was allowed to adhere for 90 min. After initial adherence, the biofilms were allowed to grow for 48 h at 37°C with a flow rate 0.2 mL/min. Mature biofilms were stained with 1 μL/mL Syto 9 (green; indicates live bacterial cells) and 1 μL/mL propidium iodide. Three slides were analyzed for every condition and at least three different z-stacks were obtained per slide. Representative 3D surfaces are shown. The white line in all images indicates 50 μm. **(A)** Untreated **(B).** Lincomycin **(C)** Amoxicillin **(D)** Oxytetracycline.

**FIGURE 5:**
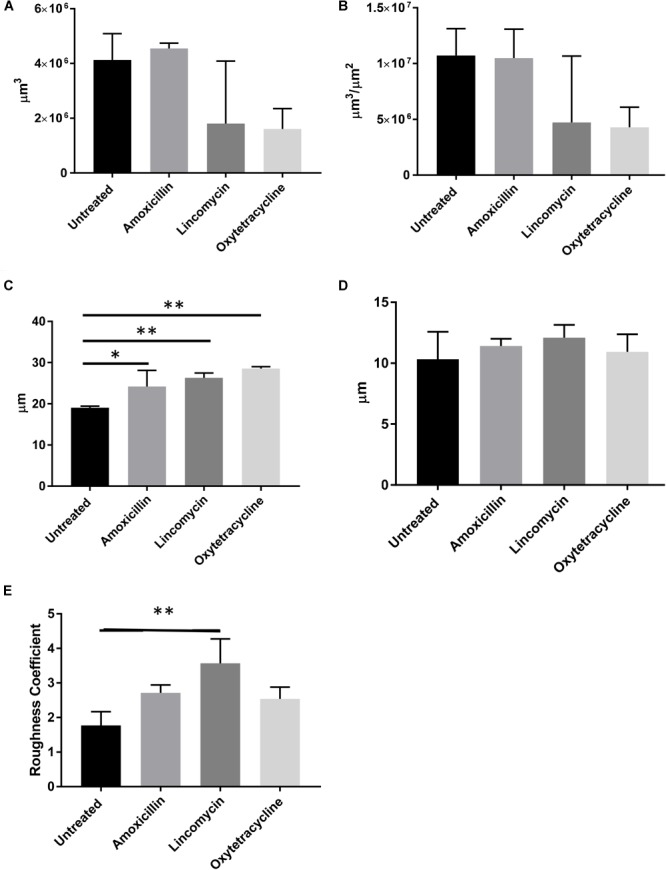
Sub-MICs of specific antibiotics increase max surface thickness and roughness of biofilm. Quantitative parameters were calculated based on live cell biofilm surface reconstructions. **(A)** Biovolume **(B)** Biomass **(C)** Maximum surface thickness **(D).** Mean surface thickness **(E)** Roughness. (*N* = 3; Ordinary one-way ANOVA with Dunnett’s multiple comparison post-test; ^∗^*p* < 0.05, ^∗∗^*p* < 0.01; Error bars indicate standard deviation).

In order to investigate the effects of antibiotics on biofilm using a flow cell biofilm assay, we utilized the highest concentration of antibiotics that demonstrated an increase in static biofilm formation without a negative effect on growth: ¼ MIC amoxicillin, ½ MIC lincomycin, and ¼ MIC oxytetracycline. *S. suis* was injected into the flow cell system and allowed to adhere for 90 min before the flow of media was started. Biofilm surfaces were constructed for all stacks for both live (Figure 4) and dead cells and were analyzed for quantitative parameters. None of the biofilms displayed any difference in biovolume, biomass, or mean surface thickness (Figures 5A,B,D). Bio-volume measures the overall volume of the biofilm based on the number of pixels present in the image (Heydorn et al., 2000), while biomass is the area of the biofilm divided by the volume. All three antibiotic treated groups exhibited an increase in the maximum surface thickness over untreated wild-type (Figure 5C) and lincomycin increased the amount of roughness in the *S. suis* biofilm (Figure 5E). The increase in maximum surface thickness and roughness is indicative of 3D-structures and architectural features that represent mature biofilms. No difference in any of the quantitative parameters upon analysis of the dead cells present in the biofilm was observed (data not shown). The increase in maximum surface thickness and roughness or irregularity of the biofilm formed by live cells can be observed in the constructed surfaces of the treated samples (Figures 4B,C,D) compared to untreated (Figure 4A).

**FIGURE 6:**
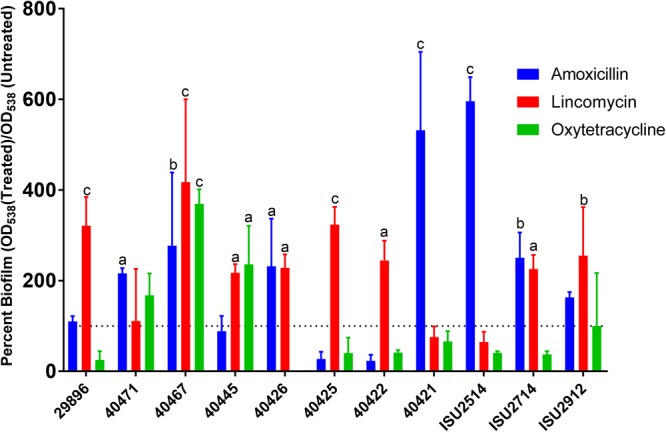
Sub-MICs of amoxicillin, lincomycin, and oxytetracyline increase biofilms formed by clinical *S. suis* strains. Eleven *S. suis* strains were incubated O/N in a 96-well plate with sub-MICs of amoxicillin, lincomycin, or oxytetracycline. After incubation, the plate was stained using a standard crystal violet assay and visualized by OD_538_. Antibiotic treated samples were compared to untreated samples to determine a percent increase in biofilm formation. Values are the means ± standard deviations (error bars) from three independent experiments, with three technical replicates in each experiment. Significant differences were assessed by ordinary one-way ANOVA with Dunnett’s multiple comparison post-test; ^a^*p* < 0.05, ^b^*p* < 0.01, ^c^*p* < 0.0001).

Using the concentrations identified as effective in increasing biofilm formation in *S. suis* strain ISU1606 (Table 1 and Figure 3), we measured the biofilm formation of an additional 11 recently acquired clinical *S. suis* isolates (Figure 6). All strains increased biofilm production when exposed to sub-MIC concentrations of amoxicillin, lincomycin, or oxytetracycline (Figure 6). Lincomycin increased biofilm production in eight of the 11 tested strains, while amoxicillin increased seven and oxytetracycline increased three.

## Discussion

In this study, we have utilized a virulent clinical swine *S. suis* isolate to measure biofilm formation. Additionally, we have demonstrated that sub-MIC concentrations of the predominantly used swine antibiotics amoxicillin, lincomycin, and oxytetracycline increase the formation of biofilms in a static biofilm assay. Interestingly, we did not see an increase in biofilm formation for nine antibiotics tested: bacitracin, carbadox, chlortetracycline, enrofloxacin, gentamicin, neomycin, sulfadimethoxine, tiamulin, and tylosin. To investigate if amoxicillin, lincomycin, and oxtetracycline would also affect biofilm formation under a steady flow environment, we utilized a flow-cell biofilm assay. The assay showed that these antibiotics also changed the *S. suis* biofilm formation with increased values of maximum height of biofilms and their roughness coefficient compared to no treatment. In order to deduce if this increase in biofilm formation is a wide-spread phenomenon, we tested an additional 11 clinical strains using our standard crystal violet assay. All 11 strains showed an increase in biofilm formation by at least one of the three antibiotics tested suggesting a more wide-spread trend.

The bacterial response to these antibiotics can be evaluated by first understanding how the changing topography of the biofilm can influence exposure to the environment. The increase in the roughness coefficient of the biofilm correlates to a change from a more homogeneous smooth surface of a biofilm to a more heterologous, irregular surface. The change in surface structure has physiological implications. Shen et al. (2015) demonstrated that an increase in *Legionella pneumoniae*’s biofilm roughness increases both the attachment of new cells to the existing biofilm and the resistance of the biofilm to detachment. The correlation between roughness and increased adherence of incoming bacteria cells was also demonstrated for *Escherichia coli* (Janjaroen et al., 2013). This increase in roughness is not limited to exposure to antibiotics but has also been shown to occur in response to environmental stress as shown for *Candida albicans* under starvation or decreased pH conditions (Ning et al., 2013; Vasconcellos et al., 2014). The larger the difference between the peaks and valleys of a biofilm, the greater the amount of roughness and maximum height of the biofilm. This leads to a decrease in the area of the biofilm under shear forces and increases the amount of biofilm that is protected from the environment. Not only can an increase in roughness aid in surviving in the environment but previous work has suggested that an increase in biofilm roughness in a common bovine pathogen, *Histophilus somni*, is correlated with an increase in pathogenicity (Sandal et al., 2007), as pathogenic strains form more rough biofilms than non-pathogenic strains.

To test whether increased biofilm formation upon exposure to sub-MIC concentrations of amoxicillin, lincomycin, and oxytetracycline is a phenotype exhibited by a single *S. suis* isolate or a broader phenotype exhibited by *S. suis* isolates in general, biofilm formation by recently acquired clinical isolates was evaluated with and without antibiotics. All recently acquired clinical *S. suis* strains displayed an increase in biofilm formation after exposure to at least one of the antibiotics tested, with the majority exhibiting increased biofilm formation after exposure to sub-MICs of amoxicillin and lincomycin. These results demonstrate that increased biofilm formation after exposure to sub-MIC concentrations of these antibiotics is a species-wide phenotype and not limited to single *S. suis* isolate. The increased biofilm formation resulting from sub-MICs of lincomycin is especially concerning given that the therapeutic dose was utilized in the biofilm assays. Thus, if a swine herd was being treated with lincomycin for respiratory symptoms, it would be feasible for *S. suis* colonizing the respiratory tract of a member of the herd to be potentially exposed to sub-MICs of lincomycin that could lead to increased biofilm formation.

Sub-MICs of antibiotics from diverse classes and thus different modes of action can induce similar alterations in a variety of bacterial phenotypes such as virulence, biofilm formation, quorum sensing, gene expression and gene transfer (Davies et al., 2006; Andersson and Hughes, 2014). To add an additional layer of complexity, sub-MICs of antibiotics from the same class have been reported to induce different changes in bacterial phenotypes (Goh et al., 2002; Tsui et al., 2004). These varied outcomes result from sub-MICs of antibiotics affecting a range of cellular processes by functioning as signaling molecules, influencing levels of the alarmone ppGpp (guanosine tetraphosphate), changing nutrient usage, and/ or inducing the SOS response (Goh et al., 2002; Davies et al., 2006; Andersson and Hughes, 2014). The activation of mobile genetic elements resulting in horizontal gene transfer by sub-MICs of antibiotics has been described (Meessen-Pinard et al., 2012; Scornec et al., 2017). Future studies are underway to investigate the transfer of antimicrobial resistance elements in biofilms formed during exposure to sub-MICs of antibiotics.

The gap in our understanding of the mechanisms that contribute to the chronic asymptomatic carriage of *S. suis* in the respiratory tract of pigs severely limits the development of vaccines and other intervention strategies. Our study begins to address this lack of information by highlighting both the need of vaccines and intervention strategies that can decolonize *S. suis* from the respiratory tract as well as choosing the most appropriate antibiotic when treating bacterial infections in swine. To summarize, our study is the first to examine the effects of sub-MICs of a variety of commonly used antibiotics in the swine industry on the formation of biofilms by *S. suis*. Collectively, the data reported here can be utilized by veterinarians in determining the most appropriate antibiotic to be used as a treatment for swine disease while limiting unintended collateral effects.

## Author Contributions

UW and TN conceived and designed the experiments, analyzed the data, contributed reagents, materials, analysis tools and wrote the paper. UW performed the experiments. All authors gave approval of the final version to be published and agreed to be accountable for all aspects of the work.

## Conflict of Interest Statement

The authors declare that the research was conducted in the absence of any commercial or financial relationships that could be construed as a potential conflict of interest.
